# MR Quantification of Total Liver Fat in Patients with Impaired Glucose Tolerance and Healthy Subjects

**DOI:** 10.1371/journal.pone.0111283

**Published:** 2014-10-24

**Authors:** Zhi Dong, Yanji Luo, Zhongwei Zhang, Huasong Cai, Yanbing Li, Tao Chan, Ling Wu, Zi-Ping Li, Shi-Ting Feng

**Affiliations:** 1 Department of Radiology, The First Affiliated Hospital of Sun Yat-Sen University, Guangzhou, China; 2 Department of Radiology, The University of Texas Southwestern Medical Center, Dallas, Texas, United States of America; 3 Department of Endocrinology and Diabetes Center, The First Affiliated Hospital of Sun Yat-sen University, Guangzhou, China; 4 Medical Imaging Department, Union Hospital, Hong Kong; 5 Department of Radiology, Kiang Wu Hospital, Macao; Scientific Directorate, Bambino Hospital, Italy

## Abstract

**Objective:**

To explore the correlations between liver fat content and clinical index in patients with impaired glucose tolerance (IGT) and healthy subjects.

**Materials and Methods:**

56 subjects were enrolled and each of them underwent upper-abdominal MRI examination that involved a T1 VIBE Dixon sequence. 14 was clinically diagnosed with IGT (collectively as IGT group ) while 42 showed normal glucose tolerance,(collectively as NGT group). NGT group was further divided into NGTFat (BMI≥25, 18 subjects) and NGTLean (BMI<25, 24 subjects). The total liver fat contents was measured and compared with clinical findings and laboratory results in order to determine statistical correlations between these parameters. Differences among IGT, NGTFat and NGTLean groups were evaluated.

**Results:**

For all the subjects, fat volume fractions (FVFs) ranged from 4.2% to 24.2%, positive correlations was observed with BMI, waist hip ratio(WHR), low density lipoprotein(LDL), fasting plasma insulin(FPI), homeostasis model assessment insulin resistance (HOMA-IR) and homeostasis model assessment β(HOMAβ). FVFs of IGT group (p = 0.004) and NGTFat group (p = 0.006) were significantly higher than those of NGTLean group.

**Conclusions:**

People with higher BMI, WHR and LDL levels tend to have higher liver fat content. Patients with BMI≥25 are more likely to develop IGT. Patients with higher FVF showed higher resistance to insulin, thus obtained a higher risk of developing type 2 diabetes mellitus.

## Introduction

Ectopic fat deposition is commonly seen in many diseases, especially in metabolic disorders, such as obesity, insulin resistance, and other components of metabolic syndrome. It plays an important role in the development and progression of nonalcoholic fatty liver disease (NAFLD) [1]–[3]. It is thought to have direct positive impact on the increasing rate of obesity and NAFLD prevalence across the world [4]. According to some researchers, about 10∼20% patients with NAFLD are affected by the progressive form of NAFLD, otherwise known as nonalcoholic steatohepatitis (NASH) where 20∼30% of patients with NASH will develop cirrhosis and end-stage liver disease [5]–[7].

Liver fat content is an essential indicator for the progression of NAFLD [8]. So far, many techniques and methods have been developed for diagnosing fatty liver disease and quantifying liver fat content. This can be achieved by techniques such as liver biopsy/pathology, computed tomography (CT) scanning, ultrasound, and magnetic resonance imaging (MRI). However, all these techniques have various shortcomings. For example, the gold standard for assessing fat content in liver, biopsy/pathology, is invasive and subjective to sampling error [9]–[12]. CT can only be used to detect the existence of fat but not to measure the total amount of the fat content. Furthermore, the accumulative radiation exposure from repeated CT scans can be harmful which limits its role in evaluating disease progression during long term follow up. In comparison, Magnetic resonance (MR) spectroscopy has been widely-applied and can provide accurate evaluation of hepatic fat content, however, it is time consuming and evaluation only limits to part of the liver [13]–[15].

MR technique for quantifying fat content is based on chemical shift imaging. It is one of the most promising techniques due to its non-invasive, radiation-free and easy-to-perform characteristics [14], [16]. Dixon technique is a two-point chemical shift-based fat-water separation method described by Dixon in 1984 [17]. It acquires two images with different echo times (TE) to separate the fat signals from the water signals in the same voxel (in-phase and out-of-phase). Pure fat image and water image are obtained by the following equation:
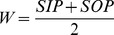





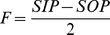



(W: water phase; F: fat phase; SIP: signal of in-phase; SOP: signal of out-of-phase).

With this technique, the map of fat deposition in the liver can be displayed, and the fat content across the entire liver can be measured.

Clinical and laboratory parameters such as body mass index (BMI), waist hip ratio (WHR), serum triglycerides (TG), and low density lipoprotein (LDL) levels are thought to be related to extent of fat accumulation in liver [18], [19]. Accurate fat quantification of the entire liver, and the investigations of the relationship between liver fat content and clinical parameters may provide information for future clinical interventions. This study aims to validate the use of Dixon technique in total liver fat content quantification, as well as to explore the correlations between liver fat content and clinical paremeters in both healthy subjects and those with impaired glucose tolerance.

## Materials and Methods

### Ethic statement

The study was conducted in accordance with ethical guidelines for human research, the Health Insurance Portability and Accountability Act (HIPAA) and was approved by the research ethical committee of The First Affiliated Hospital of Sun Yat-sen University. Written informed consent was obtained from all patients, and legal guardian if the patients were younger than 18 years old. Permission was obtained from the hospital for the publications of medical images seen in the Figures.

### Phantom validation

**Figure 1:**
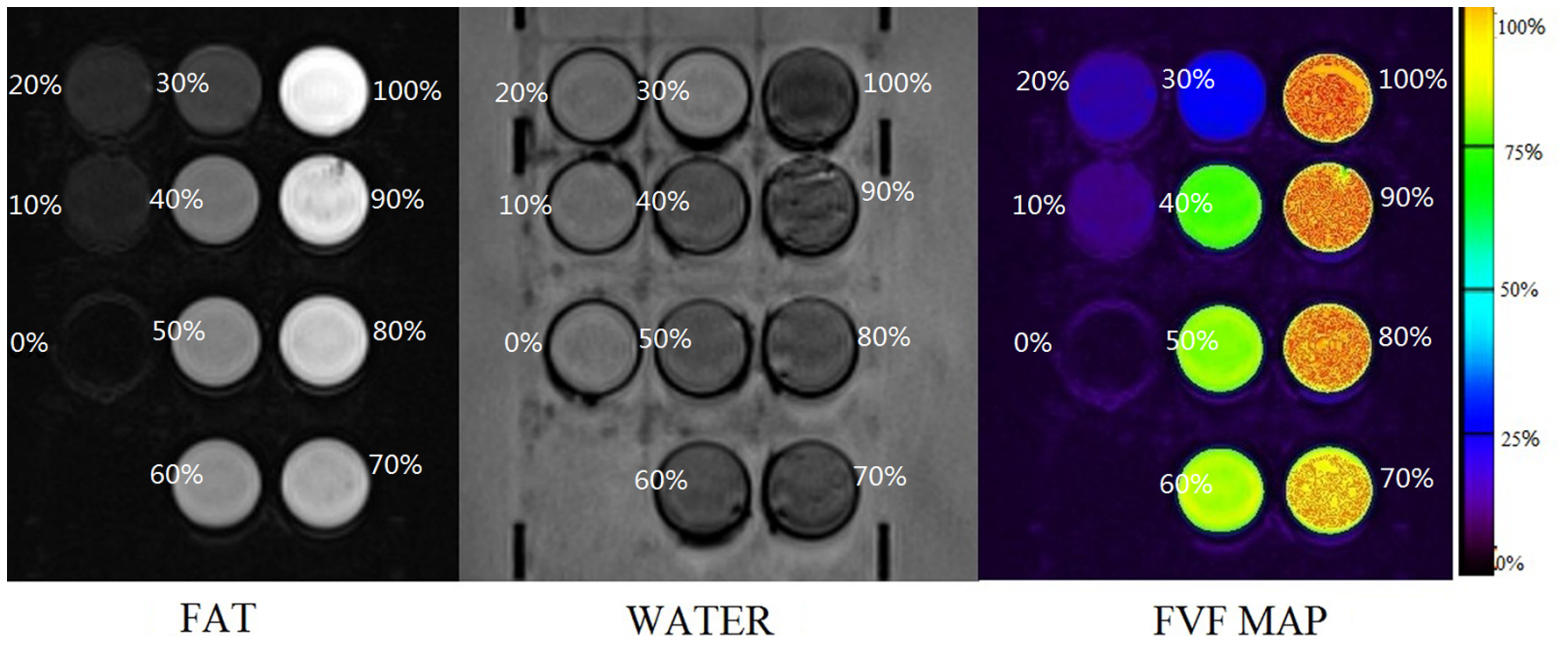
Results of phantom experiment. The bottle on the lower left side is filled with pure water (fat content: 0%), and the upper right one is filled with 100% peanut oil, while others have fat content ranging from 10% to 90% in sequence. In the fat phase image, the higher the fat content is, the higher signal intensity can be seen. But in water phase, the signal intensity of each bottle turned opposite to that of fat phase. The fat volume fraction (FVF) map demonstrates clear differences for different amounts of fat and water through the change of color.

We first evaluated the accuracy of the fat-quantifying technique with some fat-water phantoms. Homogeneous emulsions consisting of vegetable (peanut) oil and distilled water were prepared in 50 ml bottles, with fat volume fractions (FVF) of 0%, 10%, 20%, 30%, 40%, 50%, 60%, 70%, 80%, 90%, 100%, using a magnetic stirrer hotplate heated to 50°C. Agar gel (2% by weight) and dioctyl sulfosuccinate sodium salt (15 mmol/L) were added in each bottle to stabilize the emulsions (Figure 1) [20]. All the bottles were placed in a water tank with a temperature of 37°C (mimic the temperature of normal human body).

MR images were obtained using a 3-Tesla whole-body human MRI scanner (SIEMENS 3.0T MAGNETOM Verio). A head coil was used and T1 volumetric interpolated breath-hold examination (VIBE) Dixon sequence was applied with the following parameters: TE, 2.5 ms, 3.7 ms; TR, 5.62 ms; flip angle, 5°; receiver bandwidth, ±504.0 kHz; slice thickness, 3.0 mm. Four images of the phantoms were generated, including in-phase and out-of-phase, as well as fat and water phase images.

### Subjects and clinical data

Fifty-six subjects were enrolled in the study. Oral glucose tolerance test was conducted subjects were divided into either impaired glucose tolerance (IGT) group or normal glucose tolerance (NGT) group accordingly. 14 were clinically diagnosed with impaired glucose tolerance (2 males, 12 females, age range 47∼58 years old, average age was 53 years old) and 42 with normal glucose tolerance (18 males, 24 females, age range 43∼57 years old, average age 50 years old). Other clinical parameters and laboratory tests were also measured: BMI, WHR, body fat content (BFC), total cholesterol (CHOL), TG, LDL, fasting plasma insulin (FPI), homeostasis model assessment IR (HOMA-IR), homeostasis model assessmentβ (HOMAβ). NGT group was further divided into two groups according to BMI: NGTFat group, BMI >25 (the upper-limit in healthy people), 18 subjects (BMI: 26.6±1.4); NGTLean group, BMI <25, 24 subjects (BMI: 23.0±1.4). The BMI of all 14 patients in IGT group were higher than 25(BMI: 28.0±2.2).

Each subject underwent an upper-abdominal MRI examination that involved an initial set of localizer images and then a T1 VIBE Dixon sequence. The imaging parameters were the same as those in the phantom study, with TE of 2.5/3.7 ms and TR of 5.47 ms. All the subjects were imaged in supine position and carefully instructed to breath hold during end inspiration to ensure consistency amongst subjects. Four images, in-phase, out-of-phase, fat-phase and water-phase, were obtained at the same time within a single breath-hold for each patient.

### MR Image post processing and analysis

Fat volume fraction (FVF) map was generated by combining the MR images obtained during both fat-phase and water-phase of each subject. This was done by using a newly designed plug-in algorithm created under MATLAB platform (MATLAB r2011b, MathWorks, America), using the following equation:
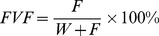



(FVF: fat volume fraction; F: fat volume; W: water volume).

In phantom study, two trained radiologists manually traced a round-shaped region of interest (ROI) on each bottle, and recorded the results of FVFs. The diameter of ROI is 3 cm, the size of the transverse section of the bottles. Average FVFs of each bottle were acquired.

**Figure 2:**
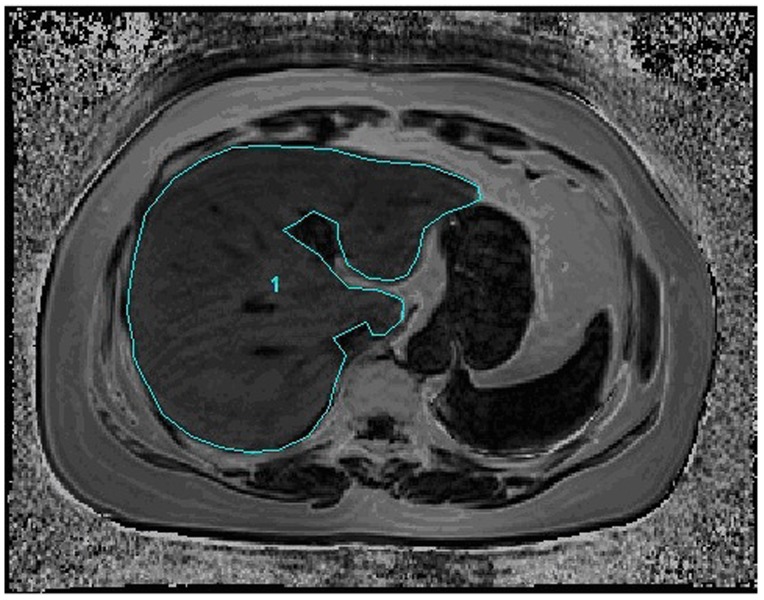
Irregular-shaped Regions of Interest (ROI) (1) covering the entire liver was placed in 21 consecutive slices (max-area centered) on the fat volume fraction (FVF) map of a typical patient. The average FVF in the ROI was recorded.

In vivo study, two trained radiologists manually placed an irregular-shaped ROI covering the entire liver in 21 consecutive slices (max-area centered) of each patient (Figure 2). The mean FVF in the ROI of each subject was recorded.

### Statistical analysis

FVF values were calculated using the Dixon technique and compared to true FVFs in the phantom experiment using linear correlation. In vivo study, statistical correlations between FVFs and results of clinical and laboratory tests were determined by using the Spearman’s rank correlation. The difference in FVF between three groups was evaluated using the Wilcoxon test. A p value lower than 0.05 was considered as statistically significant. All the analyses were performed in SPSS (SPSS, Version 13.0, Chicago, IL, USA).

## Results

### Phantom study

**Figure 3:**
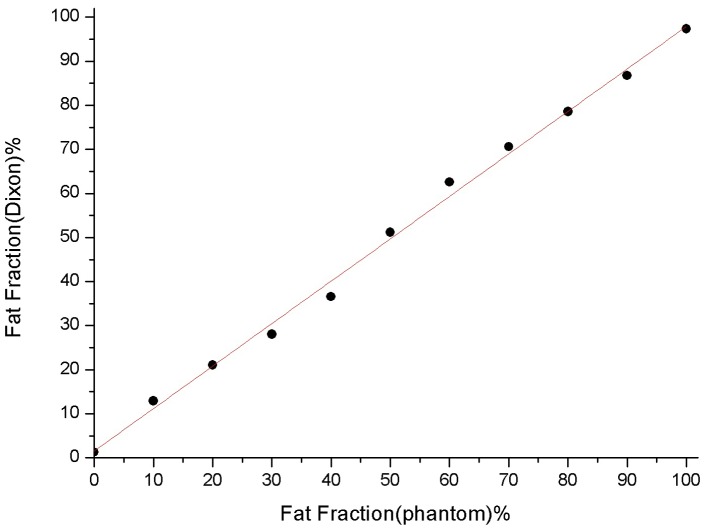
Correlation of actual fat volume fraction (FVF) of the water/fat phantoms against the mean fat fraction computed from Dixon technique. The best-fit-line was displayed. (r2 = 0.994, p = 0.000).

Figure 1 summarizes the results from the phantom study. The bottle on the lower left side is filled will pure water (fat content: 0%), and the upper right one is filled with 100% peanut oil. Others contain fat content ranging from 10% to 90%. During the fat phase image, higher fat content was associated with a higher signal intensity. The opposite was observed during the water phase image where higher fat content was associated with a lower signal intensity. FVF map demonstrated different amounts of water/fat in the emulsions ranging from 0% to 100% with changing color. A graph comparing the actual FVF against fat fraction calculated by Dixon technique also showed excellent correlation (Figure 3, r2 = 0.994, p = 0.000)). This result indicates that Dixon technique is applicable for measuring the actual fat content of the liver.

### *In vivo* results

**Figure 4:**
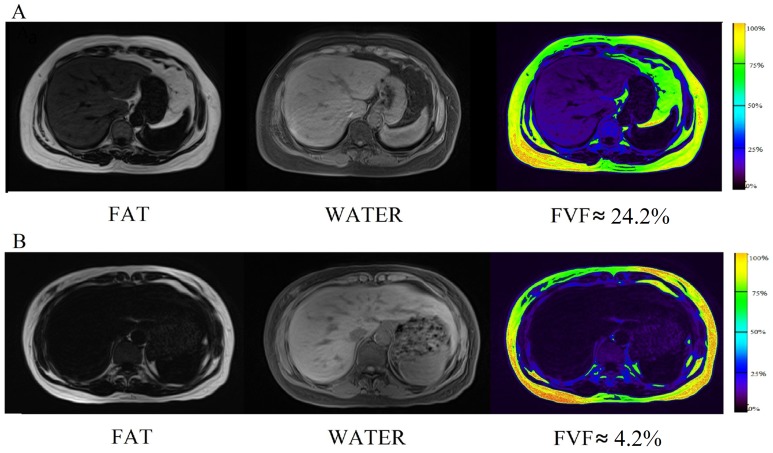
FVF maps of two typical subjects from IGT group (A) and NGTLean group (B). A: a 58 years old female who was diagnosed as IGT (BMI = 31.85), with a mean FVF of 24.23%. The fat phase and FVF map demonstrated that the deposition of fat is homogeneous. B: a 50 years old female (BMI = 23.13) with a mean FVF of 4.15%. The color of the liver in case B is much darker than that in case A, indicating a lower fat content.

For each subject, FVF maps were generated from the source images of both fat phase and water phase. Figure 4 shows the FVF maps of two typical subjects from IGT group (A) and NGTLean group (B) respectively. The darker color of the liver seen in Figure 4B indicates lower fat content in NGTLean group when compared to the IGT group as seen in Figure 4A. Furthermore, in both fat phase image and FVF map (Fig. 4A), the distribution of fat in liver in people with metabolic disorders tended to be homogeneous, which is different from fat accumulation caused by inflammation [21].

For all the subjects, FVFs ranged from 4.2% to 24.2%, and had positive correlation with BMI (p = 0.004), WHR (p = 0.045), TG (p = 0.019), LDL (p = 0.033), FPI (p = 0.019), HOMA-IR (p = 0.019) and HOMAβ (p = 0.005) (Table 1, Spearman’s rank correlation). For IGT group, FVFs positively correlated with FPI (p = 0.019), HOMA-IR (p = 0.019), HOMAβ (p = 0.005), while the FVFs of healthy groups did not show significant correlation with any of these clinical parameters.

**Table 1:** Correlations between the liver FVF values and clinical parameters in all subjects as determined by Spearman correlation test.^a.b^

Variables	Correlation Coefficient	*P*
BMI	0.595	0.004
BFC	−0.024	0.915
WHR	0.432	0.045
CHOL	0.352	0.108
TG	0.689	0.019
LDL	0.455	0.033
FPI	0.886	0.019
HOMA-IR	0.886	0.019
HOMAβ	0.943	0.005

a FVFs have positive correlation with BMI, WHR, TG, LDL, FPI, HOMA-IR and HOMAβ, while BFC and CHOL were found to have no statistical correlation with liver fat content.

b FVF, fat volume fraction; BMI, body mass index; BFC, body fat content; WHR, waist hip rate; CHOL, cholesterol; TG, triglyceride; LDL, low density lipoprotein; FPI, fasting plasma insulin; HOMA-IR, homeostasis model assessment insulin resistance; HOMAβ, homeostasis model assessmentβ.

**Figure 5:**
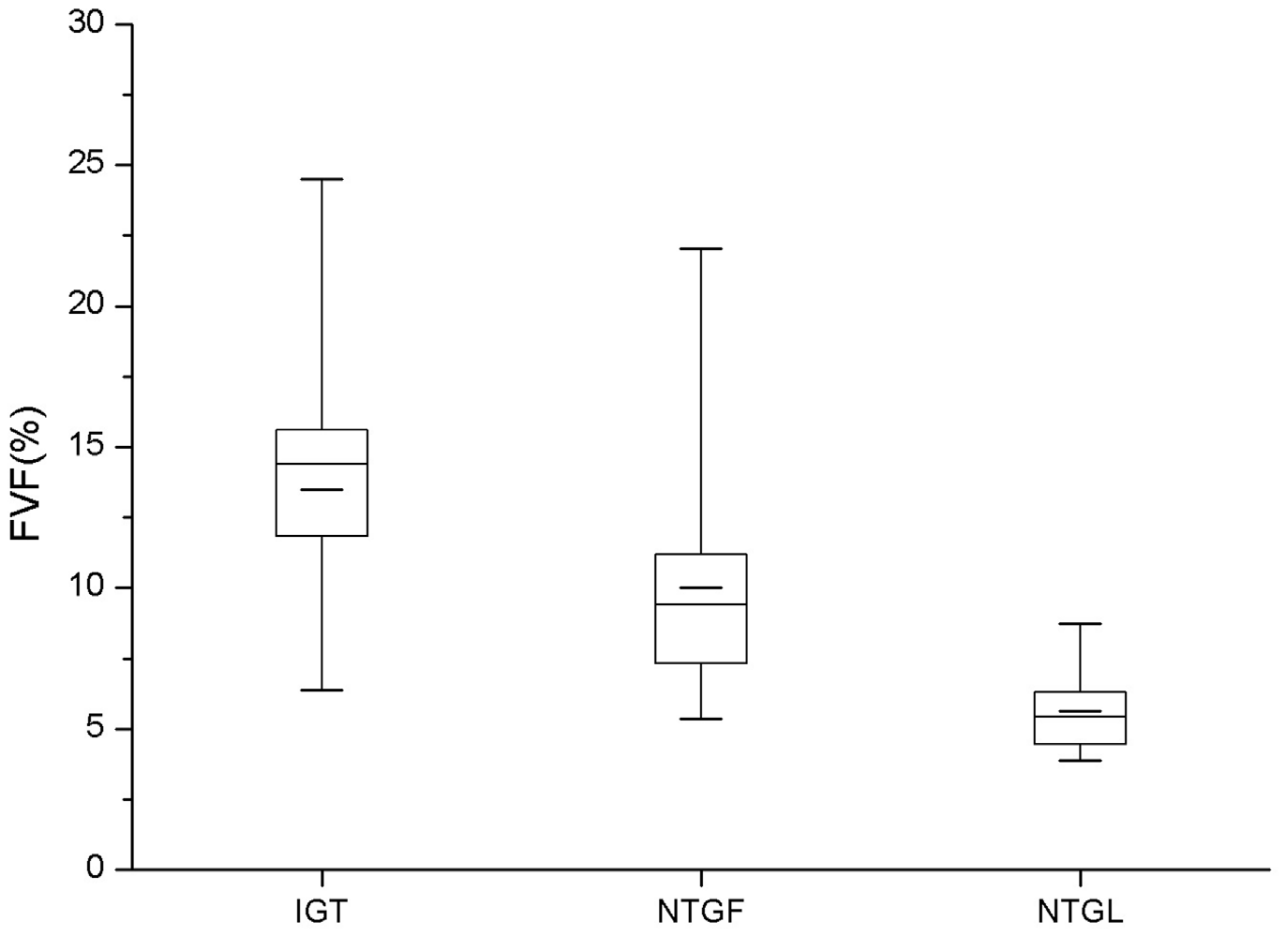
Boxplots of FVF values for the IFT, NGTFat and NGTLean groups. Significant differences was found between IGT group and NGTLean group (p = 0.004), as well as the NGTFat group and NGTLean group (p = 0.006). But there was no significant difference between IGT group and NGTFat group (p = 0.359).

FVFs of IGT group were significantly different from those of NGTLean group (p = 0.004), but no significant difference was found between IGT group and NGTFat group (p>0.05). FVFs of NGTLean group were also significantly different from those of NGTFat group (p = 0.006) (Figure 5).

## Discussion

The amount of the liver fat content is essential in monitoring the progression of NAFLD and it acts as a potential indicator of underlying metabolic disorders [22]. Unlike MR spectroscopy, Dixon technique provides us with separate fat and water phase images within a single breath-hold. Dxion technique is easier to perform than MRS; and the image analysis is also more straightforward. Liver fat content measurement and detection using Dixon technique have been reported in previous studies [23]–[27]. For example, D’Assignies, G. et al. have evaluated the ability of MRS, Dixon technique and diffusion-weighted MR imaging (DWI) to detect liver steatosis using histopathology as a reference [23]. They have also evaluated the correlations between fat fraction (FF) measured by MRS, Dixon technique and clinical parameters such as LDL and cholesterol concentrations, which showed consistent result with our study. However, their study mainly focuses on the sensitivity, specificity and accuracy in diagnosis of liver steatosis for three different noninvasive MR techniques, MRS, Dixon technique and DWI. In another study, Houchun H. Hu and colleagues have demonstrated a strong potential for IDEAL technique (Iterative Decomposition with Echo Asymmetry and Least squares estimation, a modified Dixon technique) as a liver fat quantification tool and a valuable method for obesity research [24]. However, in the studies mentioned above, only a small oval- or square-shaped ROI on the liver was studied, hence could not appropriately evaluate the total amount of the liver fat content.

In this study, from the separate fat and water phase images, a 3D voxel-wise fat fraction map of the entire abdomen was generated which showed the spatial pattern of ectopic fat, and mean FVF of the entire liver on the map was calculated. For each subject, 21 consecutive slices (max-area centered) of the FVF map was used to generate the liver ROI, and the mean value was recorded to stand for the FVF of this subject. Proved to be dependable by the phantom study, FVFs obtained via the Dixon technique is found to approximately equate to the actual fat content throughout the liver. The spatial information of the liver fat is also readily available (Fig. 1 and Fig. 4).

Under normal circumstance, liver plays an important role in the lipid metabolism. It is involved in free fatty acid uptake from the blood to synthesize TG, essential in the lipoprotein transportation by combining different types of lipids with lipid-carrying protein. When the efficiency of TG synthesis exceeds the transporting capacity of lipoproteins, TG and other types of lipids accumulates in the liver, resulting in fatty liver, or otherwise known as, ectopic fat deposition [1], [2]. Unlike fat accumulation resulted from inflammation [21], people with metabolic disorders tend to have homogeneous fat deposition, and this is demonstrated in the FVF maps. Fat accumulation caused by acute hepatitis, for instance, demonstrates focal fat content from local hepatocyte necrosis and proliferation of fibrous connective tissue. However, fat accumulation due to metabolism disorders is a systematic process, and has no region-specific predisposition. Therefore, the possible cause of liver fat deposition may be diagnosed based on the pattern of fat distribution seen in FVF map.

In our study, it was found that FVFs have positive correlations with many clinical and laboratory results such as BMI, WHR, TG and LDL, which are consistent with some previous reports [18], [19]. Besides, FVFs in IGT group is significantly higher than those of NGTLean group, while there is no significant difference seen between IGT group and NGTFat group. This indicates that fat people (BMI≥25) tend to have a higher fat content in liver due to increased burden on the liver from high free fatty acids in the blood. When the burden overwhelms the ability of processing fatty acids in liver, the fat will gradually accumulate in liver and this deposition may cause fatty liver diseases. In addition, high serum free fatty acid will slow down the utilization of glucose and stimulate the secretion of insulin. This leads to increased insulin resistance over time, which may eventually progress into IGT. Thus it is possible that the subjects of NGTFat group are at higher risk of developing IGT than people with normal BMI. This can be further supported by the lack of significant differences seen in lier fat content between the IGT and NGTFat subjects where BMIs of subjects in both groups were greater than 25.

On the other hand, we have found that liver fat content did not statistically correlate with BFC. One possible explanation is that high BFC does not equate to high viscera fat content, and the latter contributes much more to the liver fat content than BFC. Instead, WHR provided better reflection regarding the fat distribution of the body. People with high WHR have more fat deposition around the abdomen and in the viscera. Therefore, the higher WHR, the higher viscera fat deposition, and the higher the fat content in liver, as well.

This study also demonstrated that liver fat content has a positive correlation with FPI, HOMA-IR and HOMAβ. That means people with higher liver fat content tend to have higher FPI, HOMA-IR and HOMAβ levels. FPI reflects the body’s sensitivity to insulin. Higher FPI is associated with lower insulin sensitivity. HOMA-IR can be calculated from FPI, which reflects insulin resistance. Thus, our results may indicate that people with high HOMA-IR levels are more resistant to insulin. Insulin resistance is recognized as a main risk factor for many metabolic disorders, and it especially increases the probability to develop type 2 diabetes [28]. In our study, FVFs of IGT group was positively correlated with FPI and HOMA-IR. In other words, IGT patients with higher FVFs are more resistant to insulin, and may have a higher risk to develop type 2 diabetes mellitus. Therefore, fat content quantification using Dixon technique has its value as a prognostic tool for IGT patients.

To summarize, this study has verified an accurate, noninvasive and easy-to-perform method based on Dixon technique to quantify the fat content across the entire liver. FVF calculated with the technique reflects the actual liver fat content. The significant correlations of FVF with some other clinical parameters indicate further investigations are necessary for the epidemiology and pathophysiology of NAFLD as well as other metabolism disorders.
